# Antibiotic Stewardship Quality Improvement Project for Neonatal Sepsis in a Tertiary NICU in Lima, Peru

**DOI:** 10.1097/pq9.0000000000000884

**Published:** 2026-05-25

**Authors:** Carmen R. Dávila-Aliaga, Elina R. Mendoza Ibañez, Elsa Torres Marcos, Julia R. Hinojosa Pérez, Claudia V. Saldaña-Diaz, Andres Campaña-Acuña, Mary E. Davila Peña, Maria Gabriela Soza Bieli Bianchi, Charley G. Pariona Sulluchuco, Yulahima M. Martínez Ortiz, Milagros E. Macedo Villegas, Wendy L. Mendoza Jamanca, Eyleen E. Guibert Casós, Rafael Paucar Zegarra, Alfonso F. Pantoja

**Affiliations:** From the * Universidad Nacional Federico Villarreal, Lima, Perú; † Universidad Privada San Juan Bautista, Instituto Nacional Materno Perinatal, Lima, Perú; ‡ Instituto Nacional Materno Perinatal, Lima, Peru; § Universidad Nacional del Callao, Callao, Peru; ¶ Instituto Nacional Materno Perinatal, Lima, Perú; ∥ Universidad Nacional Mayor de San Marcos, Instituto Nacional Materno Perinatal; ** Colorado, EEUU. Colorado Permanente Medical Group, Denver, Colorado, EEUU.

## Abstract

**Introduction:**

Preterm newborns in neonatal intensive care units face high antibiotic exposure despite low culture positivity. To address this, the National Maternal and Perinatal Institute, Peru, and the Andean Health Organization launched a project to reduce neonatal antibiotic use by 20% from May 2023 to May 2024.

**Method:**

A quality improvement project was designed at the National Maternal and Perinatal Institute in collaboration with the Andean Health Organization as part of a multinational initiative. The project used key driver identification and Plan-Do-Study-Act cycles to enhance antibiotic stewardship in early-onset sepsis (EOS) and late-onset sepsis (LOS) cases. Monitoring focused on identifying cases of treatment failure, recurrence, and mortality rate. We present results on Laney p′-charts stratified by EOS and LOS for the antibiotic use rate. Newborn characteristics are presented in comparative tables by sepsis type.

**Results:**

We included 647 newborns for suspected sepsis, comprising 982 episodes. Antibiotic use decreased by 17.3% for EOS and 25.9% for LOS. In EOS, rule-out sepsis was predominant (67.2%), whereas confirmed sepsis was predominant in LOS (40.6%). EOS was more common in term infants with a birth weight of 1,500 to 2,500 g, whereas LOS was predominant in preterms younger than 32 weeks with a birth weight of 1,000 to 1,500 g. No treatment failures or early recurrent sepsis were observed after discontinuation of antibiotics. No readmissions occurred within 30 days. EOS mortality was 15.8% and LOS mortality was 20.2%, with a decline in overall mortality from 3.5 to 3.0 per 1,000 live births across semesters.

**Conclusions:**

The quality improvement project improved neonatal sepsis management, reduced antibiotic use, and optimized treatment protocols.

## INTRODUCTION

Newborns admitted to neonatal intensive care units (NICUs) frequently receive multiple medications, with antibiotics being administered to more than 70% of them. Preterm neonates are particularly vulnerable due to their immunologic immaturity, thin skin barrier, reduced humoral response, limited intestinal microbiota diversity, and exposure to invasive procedures, which increase their risk of severe infections such as meningitis, pneumonia, septic arthritis, osteomyelitis, and endocarditis. Because of this vulnerability, clinicians often initiate antibiotic therapy early and broadly, even when infection is unconfirmed.^1,2^ This approach contributes to a key challenge in neonatal care: distinguishing between early-onset sepsis (EOS), which affects 0.5–1.0 per 1,000 live births, has a higher incidence among preterm infants, and is linked primarily to maternal and perinatal factors; and late-onset sepsis (LOS), which develops after 72 hours and is often associated with nosocomial exposure, affecting 10%–25% of very low birth weight neonates in NICUs.^3^

However, despite differences in risk profiles and timing, both EOS and LOS are frequently treated empirically, and antibiotic courses often continue even after cultures return negative or sepsis is clinically ruled out,^4^ with an average treatment duration of 14 days for culture-negative EOS and 20 days for culture-negative LOS.^5,6^ This pattern contributes to avoidable antibiotic exposure, which is associated with necrotizing enterocolitis, LOS, mortality, disruption of the gut microbiome, and long-term risks such as atopy, obesity, and antimicrobial resistance.^7,8^

To monitor prescribing patterns, NICUs increasingly use the antibiotic use rate (AUR), expressed as days of antibiotic therapy per 1,000 patient-days. Persistently high AUR values in the setting of low culture positivity suggest opportunities to safely reduce use.

In alignment with global antimicrobial stewardship efforts, the Andean Health Organization (ORAS) launched a regional quality improvement (QI) initiative.^9^ Within this framework, the National Maternal and Perinatal Institute (INMP) of Peru, the country’s largest delivery center, implemented a QI project aimed at reducing antibiotic use in both EOS and LOS by 20% during 1 year (May 2023–May 2024), motivated by its high prematurity rate (16.1% annually) and the fact that infection-related neonatal mortality being the second leading cause of death.

## METHODS

### Setting and Context

The National Maternal Perinatal Institute (INMP) is a level III national reference center with 198 years of experience in perinatal care and the largest neonatal service in Peru. Annually, it averages 13,000 deliveries and reports a preterm birth rate of 16%, significantly higher than the national average of 9.5%.^10^ In 2023, neonatal sepsis emerged as the second leading cause of neonatal mortality at the institution. To address this burden, INMP developed and implemented a clinical practice guideline (CPG) for the management of neonatal sepsis in 2019.^11^ The medical staff, composed of neonatologists with at least 5 years of clinical practice, demonstrated high adherence to this CPG, with rates of 84.4% for EOS and 66.8% for LOS, as reported in a 2021 adherence evaluation.^12^

In February 2023, the INMP integrated into the ORAS antibiotic stewardship QI initiative to minimize the inappropriate administration of antibiotics for neonatal sepsis. Neonatal sepsis was defined according to the institutional standardized clinical criteria, requiring the presence of systemic signs of infection (eg, fever or hypothermia, tachycardia, respiratory distress) together with laboratory indicators of inflammation (eg, elevated C-reactive protein or abnormal white blood cell/absolute neutrophil counts), as assessed by the attending neonatologist. EOS refers to suspected or confirmed infections occurring within the first 72 hours of life, whereas LOS occurs after 72 hours of life.^13,14^ Confirmed sepsis was defined as the compatible clinical presentation, at least 1 abnormal inflammatory marker, and a positive blood culture. Probable sepsis involved risk factors with clinical symptoms compatible with sepsis and some abnormal laboratory findings, but negative blood cultures. Rule-out sepsis was defined as the presence of risk factors for sepsis, negative blood cultures after 72 hours, and the absence of clinical signs of infection. Any agent found in a newborn without sepsis symptoms was considered a contaminant.

As a standard protocol in our institution, every suspected case of sepsis is tested with a blood culture. Cerebrospinal fluid cultures were obtained in 85% of cases, unless contraindicated. Culture results were reported at 72 hours, with the highest number of positives detected within that period. The antibiotic therapy regimen at our institution follows the CPG recommendations as an institutional protocol and remains unchanged throughout the intervention. (**See Supplemental Digital Content 1**, which displays updated recommendations to the 2019 INMP neonatal sepsis guideline after a 2023–2024 QI initiative to optimize antibiotic stewardship in Lima, Peru, https://links.lww.com/PQ9/A754.)

Before the intervention, the INMP Epidemiology Department conducted institutional surveillance of antibiotic use through the Antimicrobial Stewardship Program (PROA), with no dedicated personnel assigned to the NICU. Reports showed an infection-related mortality rate of 4.4 deaths per 1,000 live births, although specific data by neonatal sepsis type were not available. Additionally, 37% of hospitalized newborns from January to March 2023 received at least 1 course of antibiotic therapy, as documented in clinical records.

### Primary Drivers

**Fig. 1.**
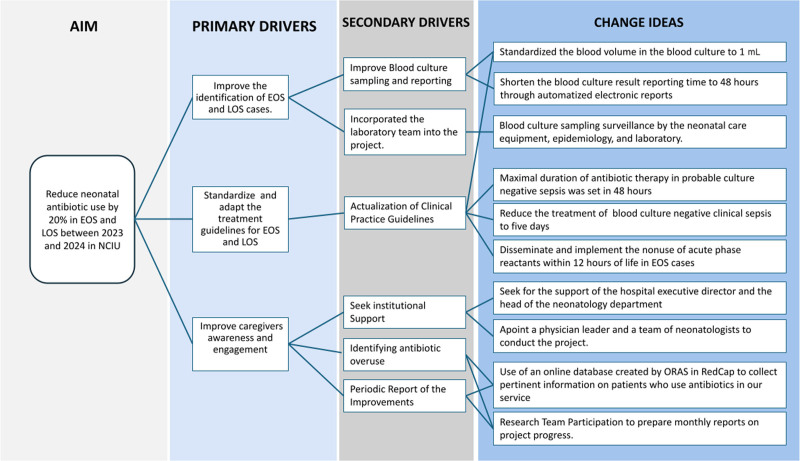
The key driver diagram helped the team identify key drivers for optimizing antibiotic treatment in EOS and LOS. These drivers focused on improving case identification, standardizing treatment protocols, and enhancing caregiver engagement, facilitating the implementation of targeted change strategies.

To identify the key factors associated with antibiotic stewardship in neonatal sepsis, the team created a key driver diagram, depicted in Figure 1. The identified primary drivers were as follows: (1) improving the identification of EOS and LOS cases, (2) standardizing and adapting the treatment guidelines for EOS and LOS, and (3) improving healthcare providers’ awareness and engagement. Addressing these factors, secondary drivers and targeted change ideas were implemented to optimize antibiotic use, minimize unnecessary administration, and improve neonatal outcomes.

### Target Population

The project targeted newborns with neonatal sepsis in the NICU from May 2023 to May 2024. We included all patients for whom a blood culture was requested due to suspected sepsis. No patients were excluded based on any clinical condition.

### Data Collection

The ORAS group provided the team with REDCap-based data collection forms, which included epidemiological and clinical records for each patient, as well as specific clinical records for each episode of EOS and LOS within each patient’s registry. A total of 8 physicians received training to complete the records through a documentary review of physical medical records and a daily review of microbiology laboratory reports, including blood cultures. The team established an EOS and LOS case alert system that included daily review of hospitalized patients’ physical medical records and laboratory results to facilitate case identification and allocation. A group of 3 expert physicians was responsible for reviewing inconsistent records. Each identified case was recorded and subsequently monitored in REDCap. At the same time, the team obtained the monthly total of patient-days in the neonatal care unit from the neonatology department’s statistics. A researcher and a biostatistician from the INMP research team generated biweekly and monthly reports on antibiotic usage trends using data recorded in REDCap.

### Measurement

The team used the AUR for EOS and LOS as a monitoring tool. The AUR represents the total number of days neonates receive antibiotic therapy, divided by the total number of patient-days in the neonatal care unit, per 1,000 patient-days. Neither the number of antibiotics used for treatment nor the number of doses administered influenced the AUR formula. In our study, we used the Laney p′ control chart to monitor biweekly changes in the AUR in the Neonatal Unit of the Instituto Nacional Materno Perinatal. This method was chosen due to the large and variable denominator—patient-days—which reflects the dynamic census of newborns at risk of antibiotic exposure. Traditional p-charts assume a binomial distribution and may yield false signals when applied to overdispersed data, a common occurrence in healthcare settings with high patient volumes and fluctuating denominators. The Laney p′-chart corrects for this overdispersion by adjusting the control limits, improving the reliability of detecting true special cause variation without overreacting to common cause variation.^15–17^ This approach is well supported in the QI literature as appropriate for monitoring proportions in complex clinical environments.^18,19^

Patient follow-up was maintained throughout hospitalization and for 1 month after discharge. Monitoring focused on identifying cases of treatment failure—defined as clinical deterioration within 7–10 days of initiating antibiotic therapy in confirmed sepsis, or within 14 days in cases of meningitis—and recurrence, defined as a new positive culture obtained at least 7–10 days after the previous one, accompanied by clinical signs of infection. Early discontinuation of antibiotics (by day 3) was based on negative blood culture results and the absence of clinical signs of sepsis. Mortality was monitored as the number of deaths caused by neonatal infection per 1,000 live births, as this was the only available baseline indicator for comparison. We also measured culture results as positive only when isolated from a patient with both clinical and laboratory findings consistent with sepsis; such cases were classified as confirmed sepsis.

### Plan-do-study-act Cycles

Following the Model for Improvement, the service implemented a series of PDSA cycles.^20^ The implemented measures were based on a critical appraisal of the best available evidence and their feasibility within our hospital setting. Table 1 presents the detailed timeline for each PDSA cycle.

**Table 1.** Timeline of PDSA Cycles

Cycle	Month	Description
PDSA 1: Identifying antibiotic overuse	April 2023	The multidisciplinary team analyzed data from the epidemiology bulletin on prior antibiotic use. A sample of 117 hospitalized patients was studied, revealing that 37% received antibiotics for a possible diagnosis of neonatal sepsis. Given the existing evidence on the risks associated with antibiotic overuse,^12,13^ the team recognized the need for stricter control of these medications. To support the improvement project, the team developed and validated a navigation chart. Subsequently, multiple meetings were held with the multidisciplinary team to provide training and standardize data collection for the database
	February 2024	Inclusion of an infectious disease physician for case evaluation and guidance on antibiotic management
PDSA 2: Prompt blood culture reporting	May 2023	A lack of knowledge of the blood culture results was identified as a contributing factor to the prolonged use of antibiotic therapy, leading to the inclusion of microbiology laboratory staff in the team. Blood culture results were printed 48 h after sample collection
	June 2023	Microbiologists tried a new approach, sharing an image of the blood culture status daily via WhatsApp. Initially, the recipients of these images were members of the QI team only
	August 2023	The images of the blood culture reports became accessible to all physicians caring for the infants in the nursery
	January 2024	The blood culture status was accessible via an electronic platform. The results were updated every 8 h
PDSA 3: Improve blood culture collection	June 2023	The multidisciplinary team coordinated meetings with the laboratory staff to share a protocol and a video presentation on best practices for blood culture collection developed by Dr. Roger Hernández, a pediatric infectious disease specialist consulted by ORAS. This protocol emphasized various aseptic techniques. The team supervised the on-site implementation of the procedure alongside the laboratory technicians
	July 2023	The neonatology and microbiology departments agreed to inject a minimum volume of 1 mL of blood into a pediatric blood culture bottle. Laboratory technicians began using 0.5% chlorhexidine wipes for skin disinfection before blood culture collection
	August 2023	The epidemiology office joined the surveillance of blood culture collection
PDSA 4: CPG modifications	July 2023	Our institution has had an evidence-based CPG for the diagnosis and treatment of early and late neonatal sepsis since 2020. In July 2023, an update to the guideline was proposed based on current scientific evidence, as recommended by our ORAS advisors as Potential Better Practices. The update was authorized by the Executive Director of Neonatology and the Head of the Department. The following modifications were implemented: • A minimum of 1 mL of blood for culture was established for suspected cases of EOS, whereas 2 blood cultures from separate sites were required for LOS • For antibiotic treatment in neonatal sepsis cases with negative blood cultures and no compatible clinical presentation, the maximum duration of antibiotic therapy was set at 48 h. In cases of persistent compatible symptoms with a negative blood culture, antibiotic treatment was extended to 5 d • To enhance the diagnostic utility of acute-phase reactants (blood count and C-reactive protein), the CPG for EOS was modified to postpone their collection until 12 h after birth The remaining aspects of the CPG remained unchanged. Compliance with the neonatal sepsis CPG and the modified guidelines is supervised by the heads of hospitalization services through a CPG adherence form, which is reviewed monthly
PDSA 5: Dissemination of AUR changes	May 2023 to September 2023	With the support of the research unit, the control charts of the AUR were presented to the team, neonatology providers, and laboratory personnel biweekly
	September 2023 to May 2024	The control charts of the AUR were presented monthly to the team, neonatology providers, and laboratory personnel throughout the project’s duration

#### PDSA Cycle 1: Identifying Antibiotic Overuse

This cycle aimed to promptly assess antibiotic use among hospitalized neonates. The implemented strategies included developing biweekly antibiotic use reports and adding an infectious disease specialist to the NICU in February 2024 to evaluate cases and provide guidance on antimicrobial management.

#### PDSA Cycle 2: Prompt Blood Culture Reporting

The objective was to reduce the turnaround time for blood culture results to 48 hours by optimizing and automating the result delivery system. The system initially began with photograph-based sharing of laboratory results to avoid delays caused by manual documentation. It later evolved into a daily Excel file containing available results, which was eventually integrated into the SIS-GalenPlus electronic medical record. Shortly thereafter, the system was automated to deliver updated laboratory results every 8 hours, surpassing the initial goal. SIS-GalenPlus is a comprehensive hospital and health information management system developed by the Peruvian Ministry of Health, with support from United States Agency for International Development and the Healthy Communities and Municipalities project. It includes an electronic health record (EHR) and manages clinical care, emergencies, and hospital admissions using modular architecture and international standards such as International Classification of Diseases, 10th Revision. The system is managed by the Ministry’s IT Office, based in Lima.

#### PDSA Cycle 3: Improving Blood Culture Collection

This cycle aimed to standardize and implement best practices for blood culture sampling by reducing false-positive cultures. The strategy included implementing 0.5% chlorhexidine wipes for skin disinfection and blood culture collection, standardizing a minimum blood volume of 1 mL in pediatric blood culture bottles, and training laboratory technicians with expert-led instruction through both theoretical instruction and hands-on practice. Asepsis compliance was ensured through regular monitoring conducted by the Office of Quality Management and the Committee for the Prevention and Control of Healthcare-Associated Infections, which submits quarterly reports to department heads.

#### PDSA Cycle 4: CPG Modifications

The objective was to update and implement evidence-based best practices in the CPG in effect at that time. This initiative was established through official communication of the new septic neonate management protocols, disseminated by the Department of Pediatrics and reinforced through internal meetings. The main updates are shown in Table 1 and **Supplemental Digital Content 1** (https://links.lww.com/PQ9/A754).

#### PDSA Cycle 5: Dissemination of AUR Changes

This cycle aimed to systematically disseminate project measurement indicators to identify trends and correct inconsistent records. The control charts were presented monthly to the entire neonatology department, the laboratory team, the epidemiology service, and the research team to raise awareness and support decision-making.

### Data Analysis

A biostatistician from the INMP research team analyzed the AUR by generating monthly and biweekly plots using annotated statistical process control charts, specifically Laney p′-charts, developed with QI Macros for Excel. The characteristics of each episode were summarized using absolute and relative frequencies for gestational age at birth, birth weight, and episode count. The analysis used the median and range (minimum and maximum values) to describe the duration of antibiotic use. Newborn characteristics were analyzed by sepsis type during EOS and LOS to assess group differences. Gestational age at birth was stratified into term (>37 wk), late preterm (32–36 wk), and very preterm (<32 wk). An independence χ^2^ test was performed.

### Ethical Considerations

The Quality Office of the INMP approved the project, ensuring compliance with established ethical standards. The team handled the data with strict confidentiality, in accordance with current regulations on the protection of personal data.

## RESULTS

Between May 1, 2023, and May 30, 2024, a total of 754 newborns were treated for 982 episodes of sepsis, of which 519 were EOS and 463 were LOS. A single episode per newborn characterized EOS cases. In LOS cases, 51% involved a single episode, 45.4% involved 2–3 episodes, and 3.7% involved more than 4 episodes.

**Fig. 2.**
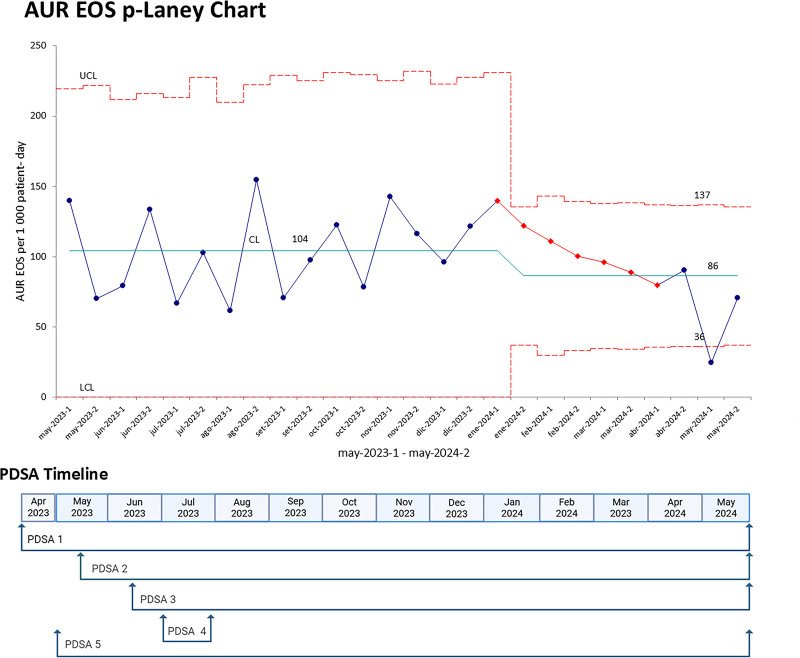
Laney p′-chart of AUR in early neonatal sepsis. Blue points represent the treatment days recorded biweekly, whereas red dotted lines indicate the upper control limit (UCL) and lower control limit (LCL); and the green line indicates the central limit (CL). Below the chart is a timeline of PDSA cycles (PDSA 1: identifying antibiotic overuse; PDSA 2: prompt blood culture reporting every 8 h through an electronic platform; PDSA 3: improving blood culture collection; PDSA 4: CPG modifications; PDSA 5: dissemination of AUR changes). The figure shows that values remained within control limits until January 2024, after which a downward trend (consecutive red points) in antibiotic use is observed (from 137 to 86), reaching a minimum AUR of 36 in May 2024. CPG, clinical practice guideline.

Among EOS cases, 3.3% were confirmed through positive blood cultures, 67.2% were ruled out as EOS, and 29.5% were probable EOS with negative cultures. Confirmed and probable EOS were most prevalent among term neonates, with the highest frequency observed in those weighing 1,500 to 2,500 g (Table 2). The p′-chart for AUR in EOS shows a 17.3% decrease from 104 to 86 days per 1,000 patient-days. A consistent downward trend is observed from the 9th month of the project’s initiation (January 2024) to the 11th month (April 2024) (Fig. 2).

**Table 2.** Characteristics of Episodes by Blood Culture Result and Type of Sepsis

Characteristic	EOS					LOS				
	Sepsis Confirmed With Positive Blood Culture, n = 17 (3.3%), n (%)	Probable Sepsis With Negative Culture, n = 153 (29.5%), n (%)	Sepsis Ruled Out, n = 349 (67.2%), n (%)	Total, n = 519 (100%)	χ^2^ (*P*)	Sepsis Confirmed With Positive Blood Culture, n = 188 (40.6%), n (%)	Probable Sepsis With Negative Culture, n = 141 (30.5%), n (%)	Sepsis Ruled Out, n = 134 (28.9%), n (%)	Total, n = 463 (100%)	χ^2^ (*P*)
Gestational age at birth					13.68 (0.0084)					6.26 (0.181)
Younger than 32 wk	5 (3.7)	41 (30.6)	88 (65.7)	134		87 (44.2)	65 (22.8)	45 (22.8)	197	
From 32 to 36 wk	3 (2.1)	25 (18.2)	109 (79.5)	137		32 (39.0)	23 (28.0)	27 (32.9)	82	
37 wk or older	9 (3.6)	87 (35.1)	152 (61.3)	248		69 (37.5)	53 (28.8)	62 (33.6)	184	
Birth weight, g					31.87 (<0.001)					13.98 (0.082)
Less than 1,000	1 (1.4)	16 (22.5)	54 (76.1)	71		46 (42.9)	37 (34.5)	24 (22.4)	107	
From 1,000 to 1,500	5 (5.2)	41 (42.7)	50 (52.1)	96		71 (46.1)	47 (30.5)	36 (23.3)	154	
From 1,500 to 2,500	6 (3.3)	56 (30.6)	121 (66.1)	183		42 (34.4)	32 (26.2)	48 (39.3)	122	
From 2,500 to 4,000	3 (1.8)	40 (24.7)	119 (73.4)	162		27 (35.1)	25 (32.5)	25 (32.4)	77	
4,000 or more	2 (28.6)	0 (0)	5 (71.4)	7		2 (66.6)	0 (0)	1 (33.3)	3	

**Fig. 3.**
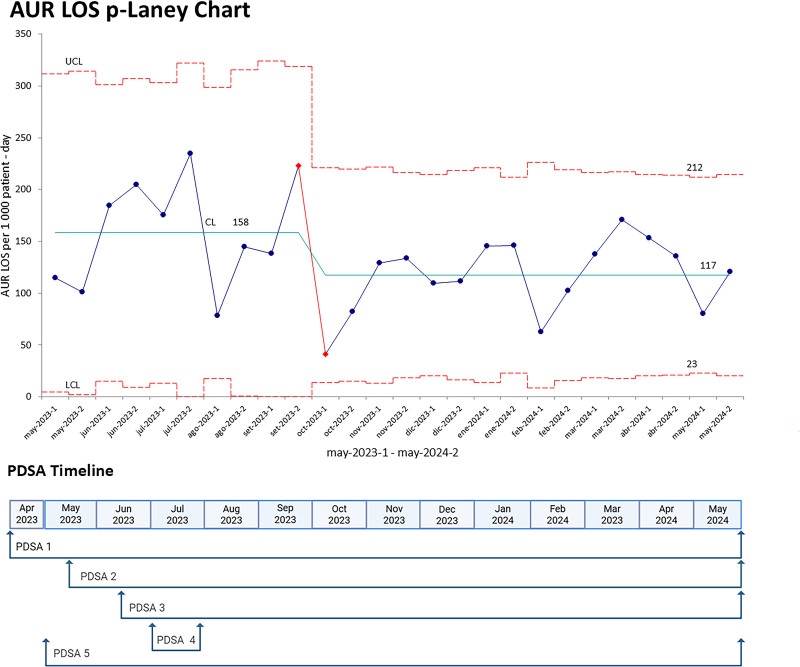
Laney p′-chart of AUR in late neonatal sepsis. Blue points represent the treatment days recorded biweekly, whereas red dotted lines indicate the upper control limit (UCL) and lower control limit (LCL); and the green line indicates the central limit (CL). Below the chart is a timeline of PDSA cycles (PDSA 1: identifying antibiotic overuse; PDSA 2: prompt blood culture reporting every 8 h through an electronic platform; PDSA 3: improving blood culture collection; PDSA 4: CPG modifications; PDSA 5: dissemination of AUR changes). The figure shows that between September and October 2023, the maximum decrease in AUR (from 212 to 23) occurred, along with a downward trend in the CL. CPG, clinical practice guideline.

In LOS cases, 40.6% were culture-positive, followed by 30.5% classified as probable LOS with negative cultures, and 28.9% as ruled-out LOS. Confirmed and probable LOS were more frequent among neonates born before 32 weeks of gestation, weighing 1,000–1,500 g, whereas ruled-out LOS was more prevalent among term neonates weighing 1,500–2,500 g (Table 2). The p′-chart of AUR in LOS showed its steepest decline from September to October 2023 (the fifth and sixth months of the project), accompanied by a reduction in the central line from 158 to 117, representing a 25.9% decrease in AUR (Fig. 3).

The most frequently isolated pathogens in EOS were unspecified Gram-positive bacteria and *Listeria monocytogenes*. The most isolated pathogens in LOS were coagulase-negative *Staphylococcus* and other *Staphylococcus* species. For details on the identified bacteria, refer to Supplemental Digital Content 2. (**See Supplemental Digital Content 2**, which displays organisms causing EOS and LOS during a QI initiative to optimize antibiotic stewardship in neonatal sepsis in Lima, Peru, https://links.lww.com/PQ9/A755.)

Regarding postantibiotic discontinuation monitoring, most of our patients were preterm neonates who remained hospitalized under close observation. None of them experienced treatment failure, and recurrent sepsis cases in those with LOS occurred more than 15 days after the previous episode. Among full-term neonates who were discharged earlier, no episodes of recurrent sepsis were identified during the remainder of their hospitalization period. Furthermore, no readmissions were recorded within 30 days of discharge, either through follow-up clinics or emergency services. As for mortality, throughout the intervention, there were 53 deaths in EOS and 70 deaths in LOS. The mortality rate during the first semester of the intervention was 3.5 deaths per 1,000 live births, and 3.0 deaths per 1,000 live births during the second semester. A total of 15.8% of newborns with EOS died, whereas 20.2% died from LOS.

## DISCUSSION

We reduced inappropriate antibiotic use for both EOS and LOS; however, it met its target of a reduction greater than 20% only in LOS, with a 25.9% decrease in AUR. Implementing 5 distinct PDSA cycles was key to achieving these outcomes. The interventions included updating the neonatal sepsis CPG in line with the best available evidence; continuously disseminating AUR data to increase provider awareness; and optimizing blood culture collection, identification, and reporting processes.

In our cohort, the proportion of ruled-out sepsis was considerably higher in EOS than in LOS, whereas confirmed sepsis cases were more frequent in late-onset presentations. This difference likely contributed to the distinct patterns of AUR reduction observed between the 2 groups. Because empiric antibiotic therapy is more commonly initiated in EOS due to nonspecific early clinical signs, timely reporting and interpretation of blood culture results had a more immediate impact in facilitating antibiotic discontinuation in EOS cases. In contrast, LOS cases often required longer treatment courses due to a higher probability of true infection; therefore, changes in prescribing behavior evolved more gradually as clinician confidence increased.

These findings underscore the importance of standardized, multidisciplinary approaches in managing neonatal sepsis. The adverse effects of antibiotic overuse in neonates are well documented, including dysbiosis, increased risk of necrotizing enterocolitis, and antimicrobial resistance.^21,22^ In response, global initiatives across various healthcare settings have demonstrated that reducing antibiotic use depends on the strategies implemented and the commitment of multidisciplinary teams in each NICU.^9,23^

One commonly adopted strategy in neonatal antibiotic stewardship is optimizing blood culture collection to improve diagnostic accuracy and reduce unnecessary antibiotic exposure. Recent studies have shown that ensuring adequate blood volume (~1 mL in suspected EOS) and obtaining 2 independent samples in LOS increase pathogen detection rates and decrease false negatives.^24,25^ Likewise, the introduction of chlorhexidine-based antisepsis has been associated with lower contamination rates and good safety profiles in preterm neonates.^26^ In our project, laboratory staff participated directly in protocol standardization and training, which facilitated consistent sample-collection practices and improved clinicians’ confidence in discontinuing antibiotics when cultures remained negative.

Regarding treatment protocols, clinicians discontinued antibiotics at 48 hours in asymptomatic neonates with culture-negative sepsis and at 5 days in symptomatic cases. This intervention reduced blood culture turnaround times by automatically uploading results to the EHR system every 8 hours. Although evidence suggests that a digital platform is not essential for timely blood culture reporting,^9^ automation has been shown to improve workflow efficiency and sustainability in NICUs.^27^ Automation also reduces delays in the availability of results and helps distinguish contaminants from true pathogens; cultures with growth beyond 18 hours are more likely to represent contamination.^28^ Other centers have used similar automation strategies to facilitate early antibiotic discontinuation, significantly reducing overall exposure.^29,30^

Multiple studies have proposed similar strategies for discontinuing antibiotics in clinically stable neonates with “culture-negative” sepsis, with varying timeframes of 48,^2,29^ 36,^31^ and even 24 hours.^23^ These approaches align with evidence that nearly all true-positive blood cultures become positive within the first 24 hours of incubation.^27,32^ Additional strategies reported in the literature include risk-based EOS calculators based on the American Academy of Pediatrics guidelines.^33^

For neonates with clinical signs suggestive of sepsis but negative blood cultures, clinicians should consider alternative diagnoses before assuming the presence of an undetectable pathogen requiring prolonged antibiotic therapy. Possible contributors include viral infections, noninfectious inflammatory conditions, localized infections without bacteremia, improper blood culture collection techniques, and prior antibiotic exposure.^5^

Regarding the use of inflammatory markers in sepsis diagnosis, C-reactive protein (CRP) remains the most used marker of inflammation. However, due to its lack of specificity and the delay in its elevation (beginning 10–12 h after infection onset),^34^ it is not recommended to initiate antibiotic therapy in EOS cases. Instead, CRP can support discontinuation of antibiotics if levels are not rising. All these measures were incorporated into our updated neonatal sepsis CPG, ensuring long-term standardization and sustainability of the interventions.

### Study Limitations

The EHR system was partially implemented during the study period and lacked a dedicated platform for recording pharmacological treatments. Consequently, data on the initiation and discontinuation of antibiotic therapy were collected manually by reviewing medical records. This process required significant effort from the research team and posed challenges for implementing continuous auditing beyond the study period. Additionally, our approach limits our ability to understand the specific, direct effect of each component because we implement the interventions simultaneously rather than sequentially. This fact represents an opportunity to address this in future interventions. Moreover, process indicators, such as rates of blood culture collection and timely antibiotic discontinuation, were not systematically collected, limiting our ability to assess how changes in practice contributed to improvements in outcomes. Future cycles will incorporate these measures to strengthen ongoing monitoring.

We consider it essential to emphasize the importance of organizational structure and context in successfully implementing a QI program. Several factors, including workload and insufficient commitment, presented significant challenges throughout this project. Evidence shows that this is a common problem to target in low- and middle-income countries.^35^

## CONCLUSIONS

In the NICU at INMP, this QI project effectively reduced antibiotic use among neonates treated for EOS and LOS. The presence of a multidisciplinary team was essential to the successful execution of interventions to optimize antibiotic stewardship. To ensure the sustainability of these measures, the automation of blood culture result uploads and the digital recording of antibiotic therapy are recommended, as these strategies facilitate antibiotic monitoring and enable timely discontinuation of treatment.

## ACKNOWLEDGMENTS

We extend our heartfelt gratitude to the multidisciplinary team of the Neonatology Department for their invaluable support and unwavering commitment to the well-being of newborns at INMP. We also sincerely thank Dr. Jaime Zegarra, Neonatologist and advisor to the Improvement Project for Peru, and Dr. Juan Manuel Grauss, responsible for the REDCap database where the data were recorded, for their mentorship throughout the execution of this research.

## Supplementary Material


