# Supplementary material for: Antibiotic Stewardship Quality Improvement Project for Neonatal Sepsis in a Tertiary NICU in Lima, Peru
Source: Pediatr Qual Saf. 2026 May 25;11(3):e884. doi: 10.1097/pq9.0000000000000884 (PMC13200947; doi:10.1097/pq9.0000000000000884)
Supplement: Supplementary file 1 [file pqs-11-e884-s001.pdf]

Supplementary material 1

Updated Recommendations to the 2019 INMP Neonatal Sepsis Guideline After a 2023–2024 Quality Improvement Initiative to Optimize Antibiotic Stewardship in Lima, Peru

Nº	RECOMMENDATION	STRENGTH OF RECOMMENDATION	UPDATED TO
1	Do not administer routine antibiotics to neonates born to mothers with PROM >18 hours as the sole risk factor.	**Strong against**<br>Very low quality of evidence	
2	Administer antibiotics to preterm neonates born to mothers with chorioamnionitis.	**Conditional in favor**<br>Low quality of evidence	
3	Do not use routine antibiotics in neonates born to mothers with fever >38 °C as the only condition.	**Strong against**<br>Very low quality of evidence	
4	Consider risk factors such as adolescence, PROM >18h, prematurity, maternal fever >38°C, UTI, and chorioamnionitis.	**Strong in favor**<br>Based on expert opinion	
5	Use ≥3 clinical signs as criteria to obtain cultures and initiate antibiotics in neonates with sepsis risk factors.	**Strong in favor**<br>Low quality of evidence	
6	Use complete blood count with leukocyte indices from 4 hours of life for suspected neonatal sepsis.	**Strong in favor**<br>Moderate quality of evidence	
7	Measure procalcitonin as an adjunct test for early diagnosis of neonatal sepsis.	**Strong in favor**<br>Moderate quality of evidence	
8	Use quantitative CRP for the diagnosis and monitoring of neonatal sepsis.	**Strong in favor**<br>Moderate quality of evidence	Postpone the collection of acute-phase reactants (blood count and C-reactive protein) for early-onset sepsis to 12 hours of life.
9	Obtain 2 blood cultures from different venous sites in neonates with suspected sepsis before starting antibiotics.	**Strong in favor**<br>Based on expert opinion	For Suspected case of Early onset sepsis, a minimum of 1 mL of blood for culture was established, while two blood cultures from separate sites were required for late-onset sepsis. Prior to blood culture collection, laboratory technicians must use 0.5% chlorhexidine wipes for skin disinfection.
10	Do not perform routine chest X-rays in neonates with suspected early-onset sepsis without respiratory symptoms.	**Strong against** (In favor only with clinical signs)<br>Very low quality	
11	Do not routinely perform urine cultures in the evaluation of early-onset neonatal sepsis.	**Strong against**<br>Low quality of evidence	
12	Do not routinely perform lumbar puncture before 72 hours in neonates with suspected early-onset sepsis.	**Strong against**<br>Very low quality of evidence	
13	Use ampicillin + aminoglycoside as the first-line regimen for both early- and late-onset neonatal sepsis.	**Strong in favor**<br>Low quality of evidence	
14	Discontinue antibiotics between 48–72 hours if blood cultures are negative and clinical course is favorable.	**Strong in favor**<br>Based on expert opinion	For antibiotic treatment in neonatal sepsis cases with negative blood cultures and no compatible clinical presentation, the maximum duration of antibiotic therapy was set at 48 hours. In cases of persistent compatible symptoms with a negative blood culture, antibiotic treatment was extended to 5 days.
15	Complete 7 days of antibiotics if blood cultures are positive and clinical evolution is favorable.	**Strong in favor**<br>Low quality of evidence	
16	Administer antibiotics for ≥14 days in neonatal meningitis based on clinical response, CSF findings, and imaging.	**Strong in favor**<br>Based on expert consensus	

For the extended version of the 2019 Clinical Practice Guideline for the Prevention, Diagnosis, and Treatment of Neonatal Sepsis from the National Maternal and Perinatal Institute of Lima, Peru follow this link: https://www.gob.pe/institucion/ins/informes-publicaciones/3612465-guia-de-practica-clinica-para-la-prevencion-diagnostico-y-tratamiento-de-la-sepsis-neonatal
