# Supplementary material for: Antibiotic Stewardship Quality Improvement Project for Neonatal Sepsis in a Tertiary NICU in Lima, Peru
Source: Pediatr Qual Saf. 2026 May 25;11(3):e884. doi: 10.1097/pq9.0000000000000884 (PMC13200947; doi:10.1097/pq9.0000000000000884)
Supplement: Supplementary file 2 [file pqs-11-e884-s002.pdf]

Supplementary material 2.
Distribution of isolated germ type by type of sepsis

GERMEN	Sepsis confirmed with positive blood culture	
	**EOS**	**LOS**
**GRAM +**	13	124
**GRAM+ Coccus**	**9**	**122**
Enterococcus faecalis	1	4
Staphylococcus spp (Coag.Neg)		33
Staphylococcus aureus	1	4
Staphylococcus auricularis		10
Staphylococcus epidermidis	1	27
Staphylococcus haemolyticus	1	41
Staphylococcus hominis		1
Staphylococcus lugdunensis		1
Staphylococcus saprophyticus		1
Streptococcus spp (Alfa-Hemolítico)	1	
GRAM Positivos, no identificados	4	
**GRAM + Bacilus**	**4**	**2**
Listeria monocytogenes	4	1
Bacilo GRAM Positivo, no identificable		1
**GRAM - bacilus**	4	**57**
Acinetobacter baumannii		3
Brevundimonas diminuta		1
Enterobacter agglomerans		1
Enterobacter cloacae		4
Enterobacter cloacae (BLEE Positivo)		1
Escherichia coli	2	6
Escherichia coli (BLEE Positivo)	1	4
Klebsiella aerogenes		1
Klebsiella pneumoniae		17
Klebsiella pneumoniae (BLEE Positivo)		4

Pseudomonas aeruginosa		1
Pseudomonas fluorescens		1
Serratia marcescens	1	10
Stenotrophomonas maltophilia		1
Bacilo GRAM Negativo, no identificable		2
**Yeast**		**3**
Candida albicans		3
**Total**	**17**	**184**
